# Two‐week prospective observational study of 5% sofpironium bromide gel in Japanese patients with primary axillary hyperhidrosis

**DOI:** 10.1111/1346-8138.16384

**Published:** 2022-04-08

**Authors:** Tomoko Fujimoto, Hiromichi Okatsu, Hiroshi Miyama

**Affiliations:** ^1^ Ikebukuro Nishiguchi Fukurou Dermatology Clinic Tokyo Japan; ^2^ Kaken Pharmaceutical Co., Ltd. Tokyo Japan

**Keywords:** 2‐week study, early effectiveness, Hyperhidrosis Disease Severity Scale score, primary axillary hyperhidrosis, sofpironium bromide gel

## Abstract

In 2020, 5% sofpironium bromide (ECCLOCK^®^) gel (hereinafter referred to as sofpironium) was approved in Japan for the topical treatment of primary axillary hyperhidrosis. A phase III study of sofpironium demonstrated the efficacy and safety of sofpironium; however, no study has assessed its early efficacy at <6 weeks after starting treatment. Therefore, to assess the earlier effectiveness of sofpironium, we conducted a 2‐week, single‐center, exploratory, prospective, observational study in Japanese patients with primary axillary hyperhidrosis. Patients aged ≥20 years and satisfying with a Hyperhidrosis Disease Severity Scale (HDSS) score of 3 or 4 at baseline were eligible for the study. The primary endpoint for the effectiveness was change in the proportion of patients with a HDSS score of 1, 2, 3, or 4 during the 2‐week study period. In 80 patients included in the full analysis set (FAS), there were more women than men (93.8% vs. 6.3%), and the mean age (±standard deviation [SD]) was 33.3 ± 9.4 years. In the FAS, the proportion of patients with a HDSS score of 1 or 2 was 55.0% on day 7, and statistically significant changes were observed after day 3 compared to baseline (*p* < 0.05). Mean HDSS scores (±SD) were significantly decreased from baseline value of 3.5 ± 0.5 to 2.4 ± 0.9 on day 7 (*p* < 0.001). The median period for sofpironium treatment to achieve a HDSS score of 1 or 2 for a continuous 2 days was 6 days (95% confidence interval, 4–8). Safety was evaluated in 92 patients in the safety analysis set, and no adverse events were reported during the study period of 2 weeks. These results suggest that after 1‐week treatment with sofpironium for patients with a HDSS score of 3 or 4, approximately 50% of the patients can achieve a HDSS score of 1 or 2, which is a clinically significant improvement for the patients.

## 1 INTRODUCTION

Primary focal hyperhidrosis is defined as a condition characterized by excessive sweating in the craniofacial, palms, soles, and axillae, through which patients have trouble in daily activities with or without heat or mental burden in the Japanese Clinical Guideline for Primary Focal Hyperhidrosis (2015 revised edition).^1^ According to an epidemiological questionnaire survey conducted in Japan from 2009 to 2010, the prevalence of primary axillary hyperhidrosis was 5.75% (334 individuals/5807 responders), and the number of patients with severe symptoms of the disease was estimated to be 2 239 000 across Japan.^2^ Therefore, many patients with primary axillary hyperhidrosis are thought to suffer from severe symptoms in Japan.

Primary focal hyperhidrosis severely affects many aspects of daily life including emotional wellbeing, interpersonal relationships, leisure activities, personal hygiene, work and productivity, and self‐esteem.^3^ In the post hoc analysis of two previous studies investigating the Short‐Form Health Survey (SF‐36) and a Hyperhidrosis Disease Severity Scale (HDSS) score of 188 patients with primary hyperhidrosis, axillary hyperhidrosis was reported to be the most common type of hyperhidrosis and had a higher negative impact on the patient's quality of life (QOL), depending on the severity of hyperhidrosis symptoms, compared with other types of hyperhidrosis.^4^ A cross‐sectional web‐based survey was conducted among 321 patients with axillary hyperhidrosis in Japan to calculate hygiene product costs and productivity loss using a work productivity and activity impairment questionnaire.^5^ In this survey, the annual hygiene product cost based on the nationwide estimation was ¥24.5 billion and the monthly productivity loss was ¥312 billion. The overall work impairment of working patients in a week was 30.52%, and the activity impairment of full‐time housewives in a week was 49.05%. The patients were often embarrassed and anxious about sweat breaking out at any moment. Therefore, it is important to reduce the symptoms as soon as possible to improve the QOL and promote social activity in patients with axillary hyperhidrosis.

Recently, a 5% sofpironium bromide (ECCLOCK^®^) gel (hereinafter referred to as sofpironium) was approved in Japan for the topical treatment of primary axillary hyperhidrosis. A phase III, 6‐week, multicenter, randomized, controlled, double‐blind, parallel‐group study of sofpironium was conducted in Japan, and demonstrated the efficacy and safety of sofpironium in patients with primary axillary hyperhidrosis.^6^ In this study, the primary efficacy endpoint, the proportion of patients who satisfied both criteria of a HDSS score of 1 or 2 and a 50% or more reduction in total gravimetric weight of sweat was significantly higher in the sofpironium group than in the vehicle group (*p* = 0.003). No serious adverse events (AE) were reported in the sofpironium group. In addition, a 52‐week, open‐label study demonstrated the long‐term efficacy and safety of sofpironium.^7^

Although the previous 6‐week phase III study indicated the efficacy of sofpironium at 2 weeks after the initiation of sofpironium treatment,^6^ to the best of our knowledge, no study has assessed its efficacy in a shorter period. Therefore, to assess the earlier effectiveness of sofpironium within the 2‐week treatment from day 1 to 14, we conducted a 2‐week prospective observational study of sofpironium in Japanese patients with primary axillary hyperhidrosis.

## 2 METHODS

### 2.1 Study design

A 2‐week, single‐center, exploratory, prospective, observational study of sofpironium was conducted in Japanese patients with primary axillary hyperhidrosis to assess the early effectiveness of sofpironium applied to the axillae once daily for 2 weeks. The safety was also evaluated. The study was conducted in compliance with the protocol approved by the Institutional Review Board of Nihonbashi Sakura Clinic held on 16 March 2021, the principles of the Declaration of Helsinki, and ethical guidelines for medical and health research involving human subjects. Written informed consent was obtained from all patients prior to their participation in the study. The study period was March to July 2021. In this study, the patients responded to the questionnaires of HDSS daily from baseline to day 14, compliance with the sofpironium treatment daily from day 1 to 14, and Dermatology Life Quality Index (DLQI) at baseline and day 14 via an internet website.

### 2.2 Study patients

Primary axillary hyperhidrosis patients who were aged 20 years or older at the time of providing informed consent, satisfying a HDSS score of 3 or 4 at baseline, and scheduled to prescribe sofpironium for the therapy of primary axillary hyperhidrosis, were eligible to participate in the study. Primary axillary hyperhidrosis was diagnosed if the patient had subjective symptoms of excessive sweating without any obvious cause that had persisted for at least 6 months at the time of informed consent, and met at least two of the following six conditions: (i) onset age of 25 years or younger; (ii) bilateral symmetrical sweating; (iii) no sweating during sleep; (iv) at least one episode of heavy sweating per week; (v) positive family history; and (vi) excessive sweating interfering with daily activities.^8^

Patients who used relevant medication/therapy before the time of informed consent (time in parentheses) were excluded from the study.

The relevant medication/therapy included: (i) herbal medicines for reducing symptoms of hyperhidrosis (within 7 days); (ii) systemic and topical anticholinergics, oral cholinergic agonists, serotonin agonists, β‐blockers, α‐adrenergic agonists, dopamine partial agonists, tricyclic antidepressants, aluminum chloride, medications for hyperhidrosis approved outside Japan, and tap water iontophoresis (within 30 days); and (iii) botulinum toxin (within 9 months).

### 2.3 Endpoints

The primary endpoint for the effectiveness was change in the proportion of patients with a HDSS score of 1, 2, 3, or 4 during the 2‐week study period. The other endpoints were the change in the mean HDSS score, cumulative achievement rate of patients with a HDSS score of 1 or 2 for continuous 2 days, and change in the mean DLQI score.

The HDSS score is used to assess the severity of primary focal hyperhidrosis by classifying subjective symptoms as follows: score 1, sweating is never noticeable and never interferes with daily activities; score 2, sweating is tolerable, but sometimes interferes with daily activities; score 3, sweating is barely tolerable and frequently interferes with daily activities; and score 4, sweating is intolerable and always interferes with daily activities. A score of 3 or 4 indicates severe hyperhidrosis, a score of 2 indicates moderate hyperhidrosis, and a score of 1 indicates absence of hyperhidrosis.^9^

The DLQI, which is designed to evaluate the skin disease‐related QOL,^10^ was modified into the DLQI (for axillary hyperhidrosis) by changing the word of “skin” in the questionnaire to “axillary”, to make it more suitable in assessment of primary axillary hyperhidrosis. Responses to 10 questions were scored and summed to obtain the DLQI (for axillary hyperhidrosis) score.

The safety endpoint was the incidence of AE assessed by the physician based on a report from patients during the study period.

### 2.4 Analysis

The target sample size was 140 patients, based on the rationale described below. In a phase III, 6‐week study of sofpironium,^6^ the proportion of patients with a HDSS score of 1 or 2 at 6 weeks was 60.3% in the sofpironium group. Assuming that the proportion in the present study would be 60.3%, the necessary number of patients was therefore calculated to be 99 with a relative precision of 0.15 with a 95% confidence interval (CI). Allowing for 30% withdrawals, the target sample size was determined to be 140 patients.

The endpoints for the effectiveness were evaluated in the full analysis set (FAS), defined as the patient population who responded to at least one questionnaire and who had a baseline HDSS score of 3 or 4, who had a ≥80% treatment compliance rate during the study period. Safety was evaluated in the safety analysis set (SAF), defined as the patient population who received at least one treatment of sofpironium.

For each endpoint, the mean, standard deviation (SD), median, minimum to maximum, and proportion (%) of patients at each assessment time point were calculated. For the endpoints for effectiveness, an observed case approach was used in which only observed values were analyzed, without imputation of missing values. The proportions of patients with a HDSS score of 1 or 2 were statistically compared between day 0 and at each evaluation point using a one‐sample proportion test. The HDSS and DLQI scores were statistically compared between day 0 and at each evaluation point using the Wilcoxon signed‐rank test and paired *t*‐test, respectively. Statistical significance was confirmed using a two‐sided alpha level of 0.05. Adjustment of multiplicity was not performed. The Kaplan–Meier method was used to assess the achievement rate of patients with a HDSS score of 1 or 2 for a continuous 2 days. R® version 3.4.0 software (The R Development Core Team) was used for data analysis.

## 3 RESULTS

### 3.1 Patient disposition

Written informed consent was obtained from 139 Japanese patients with primary axillary hyperhidrosis (male, 14; female, 125), and 92 patients completed the questionnaire (male, six; female, 86). All 92 patients were included in the SAF. Twelve of 92 patients were excluded because of a HDSS score of 1 or 2 at baseline, a <80% treatment compliance rate, and patients who completed the questionnaire within 13 days; therefore, 80 patients (male, five; female, 75) were included in the FAS.

### 3.2 Patient characteristics

In the 80 patients included in the FAS, 93.8% were female, and the median age was 33.3 years. Baseline HDSS and DLQI total scores (mean ± SD) were 3.5 ± 0.5 and 10.8 ± 4.9, respectively (Table 1).

**TABLE 1:** Baseline characteristics of study patients (FAS)

Baseline characteristics	n = 80
Age (years)	
Mean ± SD	33.3 ± 9.4
Sex, n (%)	
Male	5 (6.3)
Female	75 (93.8)
HDSS score, n (%)	
Grade 3	43 (53.8)
Grade 4	37 (46.3)
Mean ± SD	3.5 ± 0.5
DLQI total score	
Mean ± SD	10.8 ± 4.9

Abbreviations: FAS, full analysis set; DLQI, Dermatology Life Quality Index; HDSS, Hyperhidrosis Disease Severity Scale; SD, standard deviation.

### 3.3 Sofpironium treatment status

In the 80 patients included in the FAS, the median period for sofpironium treatment (range) was 14 days (12–14 days), and median treatment compliance rate was 100% (85.7–100%) during the 2‐week study period.

### 3.4 Effectiveness

#### 3.4.1 HDSS score

**FIGURE 1:**
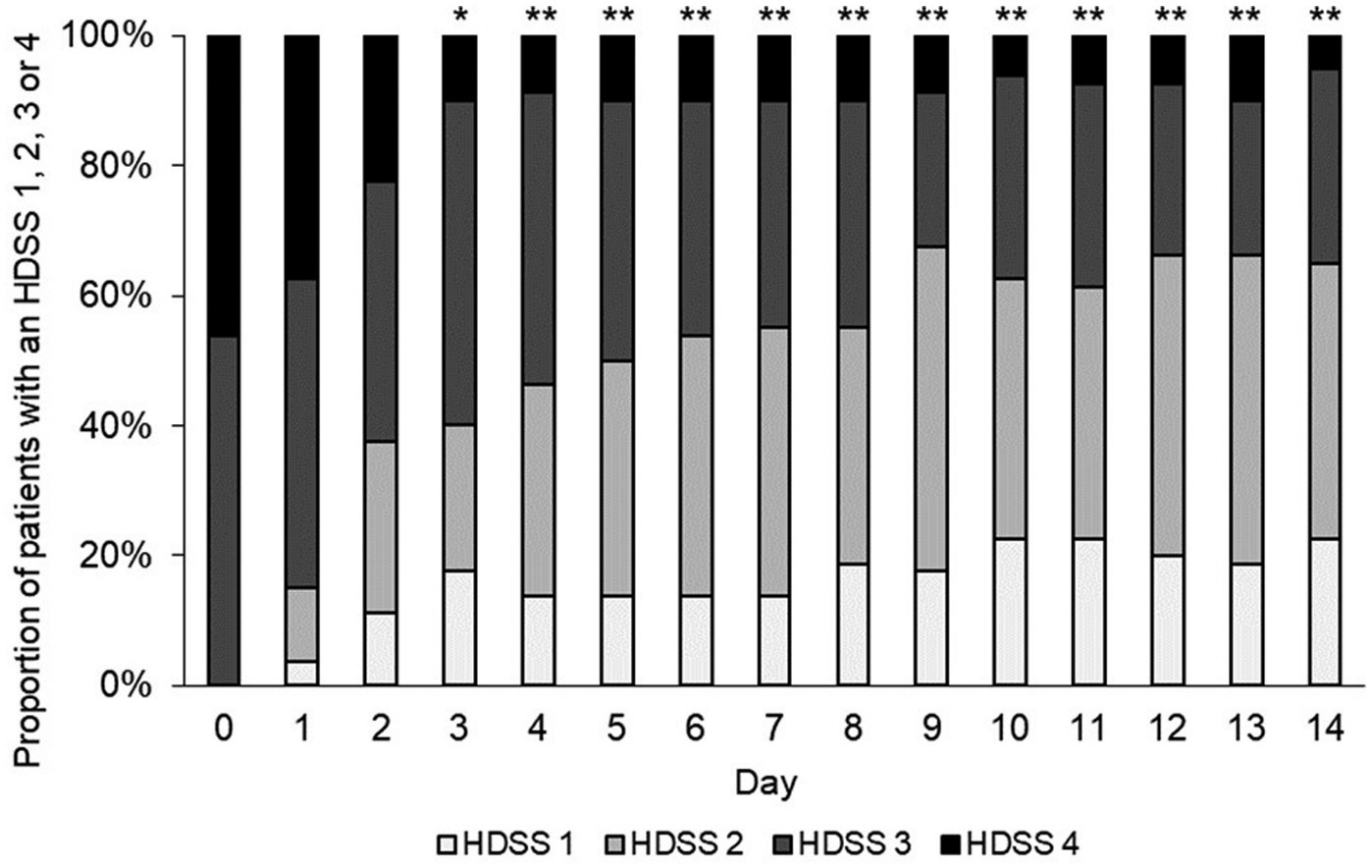
Change in proportion of patients with a HDSS score of 1, 2, 3, or 4 (FAS). The number of patients evaluated was 80 included in the FAS from day 0 to day 14. *p < 0.05, **p < 0.001 (comparison of proportion of patients with a HDSS score of 1 or 2 using a one‐sample proportion test, vs. day 0). The asterisks indicate that the proportion is significantly higher than the hypothesized value (*p* = 0.282). Abbreviations: FAS, full analysis set; HDSS, Hyperhidrosis Disease Severity Scale

In the 80 patients included in the FAS, the proportions of patients with a HDSS score of 1 or 2 were 55.0% (44/80 patients) on day 7 and 65.0% (52/80 patients) on day 14, with an increase from day 0 (baseline) to day 9, and over 50% of patients achieved a HDSS score of 1 or 2 on day 6 (Figure 1). Statistically significant changes were observed after day 3 compared to day 0.

**FIGURE 2:**
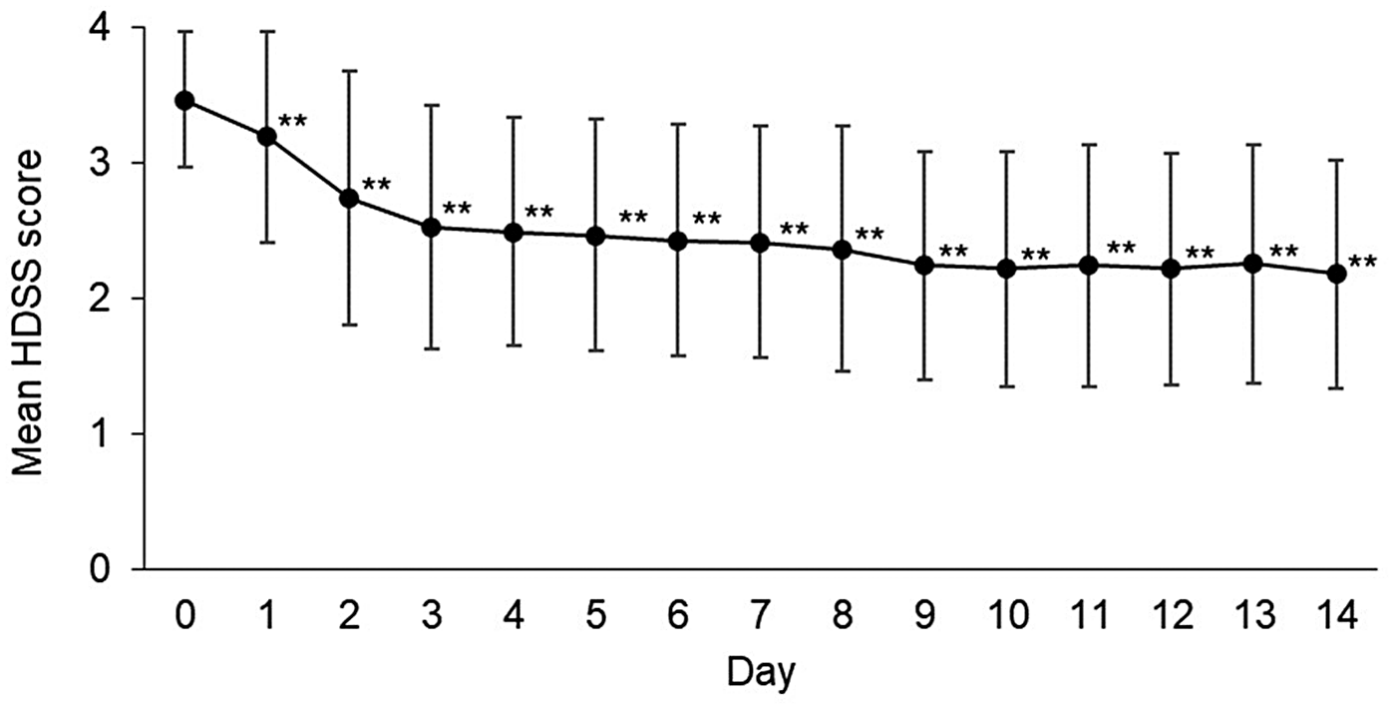
Change in the mean HDSS score (FAS). Data are expressed as mean ± SD. The number of patients evaluated was 80 included in the FAS from day 0 to day 14. **p < 0.001 (Wilcoxon signed‐rank test, versus. day 0). Abbreviations: FAS, full analysis set; HDSS, Hyperhidrosis Disease Severity Scale; SD, standard deviation

Mean HDSS scores (±SD) were decreased from baseline value of 3.5 ± 0.5 to 2.4 ± 0.9 on day 7 and 2.2 ± 0.8 on day 14, with statistically significant changes after day 1 compared to day 0 (Figure 2).

**FIGURE 3:**
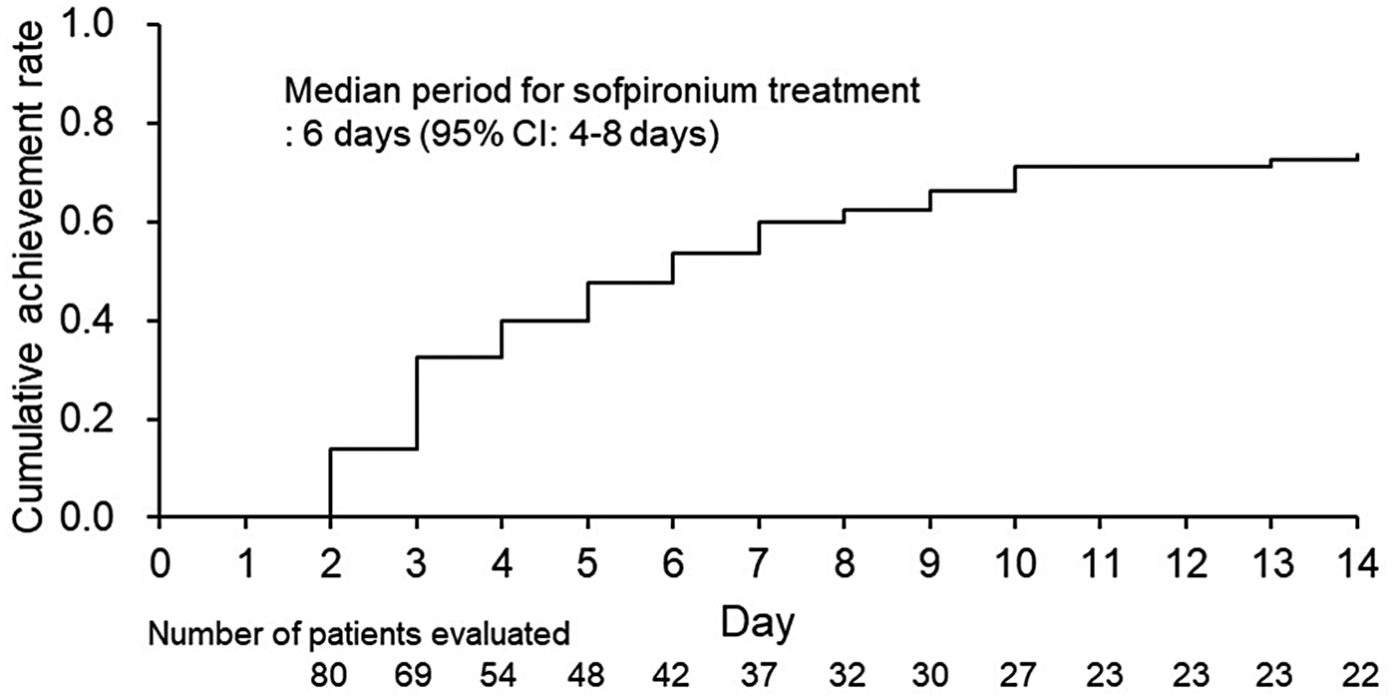
Kaplan–Meier curve of the cumulative achievement rate of patients with a HDSS score of 1 or 2 for a continuous 2 days (FAS). The median period for sofpironium treatment to achieve a HDSS score of 1 or 2 for a continuous 2 days was 6 days (95% CI, 4–8 days). Abbreviations: CI, confidence interval; FAS, full analysis set; HDSS, Hyperhidrosis Disease Severity Scale

The cumulative achievement rates of patients with a HDSS score of 1 or 2 for a continuous 2 days were 60.0% (95% CI, 49.5–70.7%) on day 7 and 73.7% (95% CI, 63.8–82.8%) on day 14. The median period for sofpironium treatment to achieve a HDSS score of 1 or 2 for a continuous 2 days was 6 days (95% CI, 4–8 days) (Figure 3).

#### 3.4.2 DLQI total score

**FIGURE 4:**
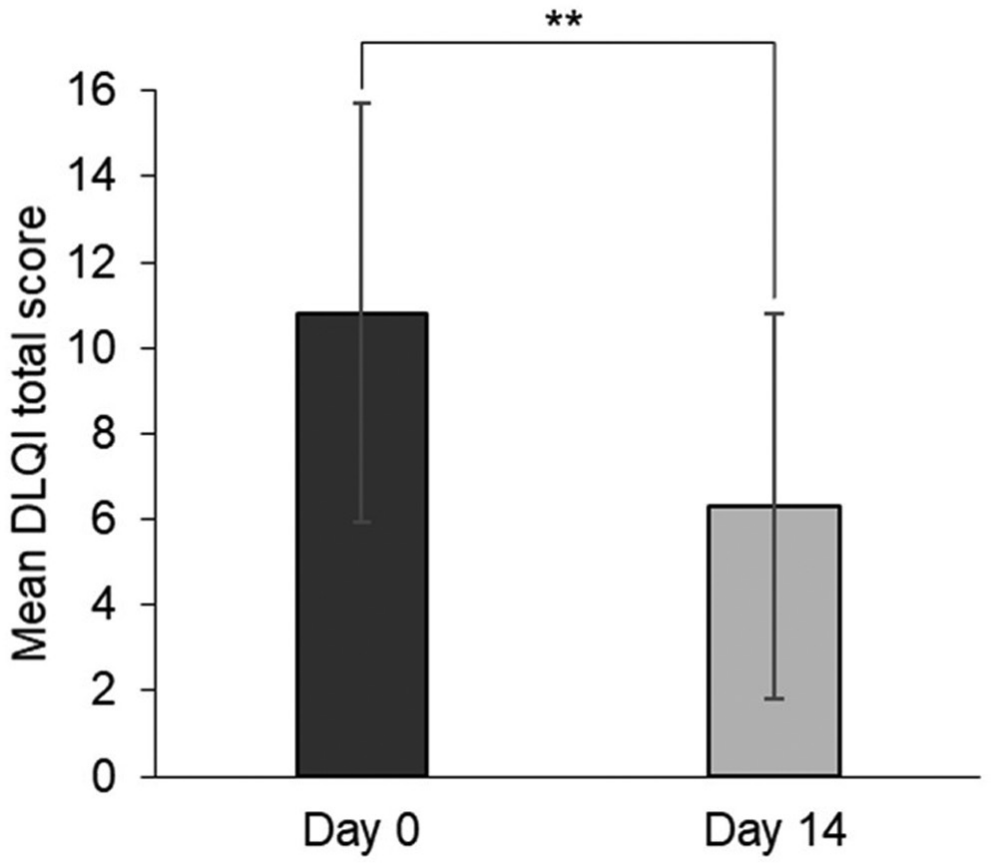
Mean DLQI total score on day 0 and day 14 (FAS). Data are expressed as mean ± SD. The number of patients evaluated was 80 included in the FAS on day 0 and day 14. **p < 0.001 (paired *t*‐test). Abbreviations: DLQI, Dermatology Life Quality Index; FAS, full analysis set; SD, standard deviation

In the 80 patients included in the FAS, the mean DLQI total scores (±SD) were decreased from a baseline value of 10.8 ± 4.9 to 6.3 ± 4.5 on day 14 (Figure 4). The mean change in DLQI total score from day 0 was −4.5 ± 4.0 on day 14, with statistical significance.

### 3.5 Safety

#### 3.5.1 Incidence of AE

Safety was evaluated in 92 patients included in the SAF, and no AE were reported during the 2‐week study period.

## 4 DISCUSSION

Primary axillary hyperhidrosis is a refractory disease that not only limits daily and social activities, but also causes psychological and emotional distress.^1^, ^8^, ^11^ In addition, this disease interferes with daily activities due to the limited choice of clothing and the requirement for frequent clothing changes or showers;^3^ therefore, improvement to tolerable sweating with a HDSS score of 1 or 2 after treatment is clinically significant. Primary axillary hyperhidrosis patients with a HDSS score of 3 or 4 were enrolled in this study. The proportion of patients with a HDSS score of 1 or 2 was significantly increased on day 3 compared to that on day 0 (*p* < 0.05), and the proportion was 55.0% on day 7. The median period for sofpironium treatment to achieve a HDSS score of 1 or 2 for a continuous 2 days was 6 days (95% CI, 4–8 days). These data demonstrated that after 1‐week treatment with sofpironium, approximately 50% of the patients achieved a HDSS score of 1 or 2, which is a clinically significant improvement for the patients.

The current Japanese guideline recommends that topical aluminum chloride should be used as a first‐line therapy for primary axillary hyperhidrosis;^1^ however, topical aluminum chloride, which is the most common topical preparation for the treatment of hyperhidrosis,^12^ has not been studied in randomized controlled studies with primary axillary hyperhidrosis patients in Japan, and is not covered by Japanese health insurance. A small scale randomized controlled study conducted in Taiwan to evaluate the efficacy and tolerability of topical aluminum sesquichlorohydrate when compared to aluminum chloride as a treatment for patients with primary axillary hyperhidrosis (n = 20), indicated that the mean response time was 1.14 weeks for both treatments.^13^ The study conditions were different in our present study and the study conducted in Taiwan, and direct comparison is therefore limited; however, sofpironium is suggested to have early effectiveness for primary axillary hyperhidrosis, which is comparable to that of topical aluminum chloride.

In the present study, the proportion of patients with a HDSS score of 1 or 2 was 65.0% on day 14. In a phase III study of sofpironium,^6^ Japanese patients with primary axillary hyperhidrosis who had a HDSS score of 3 or 4 and a 5‐min gravimetric weight of sweat per side of 50 mg or more in both axillae at baseline were eligible to participate. The proportion of patients with a HDSS score of 1 or 2 was approximately 50% after treatment with sofpironium for 2 weeks (n = 139). Patient backgrounds are different in our present study and the phase III study; therefore, direct comparison is limited; however, the effectiveness data obtained from the two studies are similar. Additionally, 55.0% and 65.0% of sofpironium‐treated patients with a HDSS score of 3 or 4 at baseline could achieve a HDSS score of 1 or 2 at 7 and 14 days after the start of sofpironium treatment, respectively in the present study. On the other hand, approximately 30% (n = 138) of placebo‐treated patients with a HDSS score of 3 or 4 at baseline could achieve a HDSS score of 1 or 2 at 2 weeks after the start of placebo treatment in the previous phase III study.^6^ These data suggest the earlier effectiveness of sofpironium within the 1‐week treatment in this study, although this study was conducted as a single (only sofpironium) arm study without a control (placebo) arm, and placebo effect cannot be ruled out.

In the safety evaluation, no AE were reported during the 2‐week study period, and no new safety signal for the sofpironium treatment was observed in this study.

Female patients with primary axillary hyperhidrosis accounted for 93.8% of 80 patients included in the FAS, although an epidemiological study reported a significantly higher prevalence of primary focal hyperhidrosis in men than in women.^2^ This single‐center study was conducted in our clinic, and most male patients with axillary hyperhidrosis who visited our clinic had head hyperidrosis as a comorbidity. These male patients received medication/therapy with oral anticholinergic agents for the treatment of head hyperidrosis, which satisfied the exclusion criteria in this study. This is the reason for the higher ratio of female patients in this study, and interpretation of the findings is needed at this point.

This study was conducted as a single‐arm, observational study; therefore, the findings from this study need to be verified via a controlled study with a control (placebo) arm.

In conclusion, a 2‐week prospective observational study of sofpironium in Japanese patients with primary axillary hyperhidrosis suggests that after 1‐week treatment with sofpironium for patients with a HDSS score of 3 or 4, approximately 50% of the patients can achieve a HDSS score of 1 or 2, which is a clinically significant improvement for the patients.

## CONFLICT OF INTEREST

This study was funded by Kaken Pharmaceutical. T.F. received fees as a resource speaker from Kaken Pharmaceutical. H.O. and H.M. were employees of Kaken Pharmaceutical. With funding from Kaken Pharmaceutical, Medical Professional Relations assisted in the writing and editing of this paper.
